# Birth weight percentiles by sex and gestational age for twins born in southern China

**DOI:** 10.1038/s41598-018-36758-6

**Published:** 2019-01-24

**Authors:** Huazhang Miao, Fei Yao, Yuntao Wu, Xiu Zhang, Rubi He, Bing Li, Qingguo Zhao

**Affiliations:** 1 Department of Healthcare, Guangdong Women and Children Hospital, No.521 Xingnan Road, Guangzhou, Guangdong 511442 China; 2 Department of Physical Examination, Guangdong Women and Children Hospital, No.521 Xingnan Road, Guangzhou, Guangdong 511442 China; 3 Department of Obstetrics, Guangdong Women and Children Hospital, No.521 Xingnan Road, Guangzhou, Guangdong 511442 China; 4 Epidemiological Research Office of Key Laboratory of Male Reproduction and Genetics (National Health and Family Planning Commission), Family Planning Research Institute of Guangdong Province, No.17 Meidong Road, Guangzhou, Guangdong 510600 China

## Abstract

Mean birth weight of twins is known to be lower than that of singletons, however, southern China lacks a twin-specific birth weight reference. In this paper, we use data from the Birth Certificate System in southern China, collected between January 1^st^ 2014 and December 31^st^ 2017 and including 161,076 twins, to calculate sex- and gestational week-specific birth weight percentiles (the 3^rd^, 10^th^, 25^th^, 50^th^, 75^th^, 90^th^, and 97^th^). We applied generalized additive models for location, scale and shape (GAMLSS) when calculating the birth weight percentiles, and calculated percentiles for monochorionic and dichorionic twins separately. We next used data collected between Jan 1st 2018 and Apr 30th 2018, encompassing 12,371 live births, to calculate the SGA and LGA ratios using birth weight references in Australia, South Korea and China (based on birth defects surveillance system) and birth weight percentiles calculated in this study. Compared to dichorionic twins, monochorionic twins had lower birth weights at 25 to 42 weeks of gestation. The calculated SGA and LGA ratios were relatively stable compared to the other references.

## Introduction

In recent years, due to the development of assisted reproductive technologies, the twin pregnancy rate continues to rise^1^. Twins have higher risks of preterm birth, perinatal morbidity and mortality^2^. Twins account for 2–4% of all infants, and the problems associated with twin pregnancies have attracted increased global attention. According to a report from the National Health and Planning Commission in China, the twin pregnancy rate increased by 4.1% in 2016^3^. Chorionicity complicates twin health further. The risk of adverse pregnancy outcomes (e.g. congenital anomalies, growth restrictions, perinatal death) and complications of fetus during pregnancy (e.g. twin-to-twin transfusion syndrome) is higher among monochorionic twins than among dichoroitic twins^4^. Therefore, chorionicity must be taken into account when establishing birth weight references for twins.

Birth weight is still the most commonly used indicator of fetal development. Infants are commonly defined as SGA or LGA if their birth weight percentile falls below the 10th percentile or above the 90th percentile of the reference standard^5,6^. SGA and LGA are associated with increased perinatal and infant mortality and morbidity, as well as long-term health problems. Twin birth weights were consistently lower than those of singletons^7^. In addition, multiple pregnancies are a risk factor associated with SGA^8^. Therefore, proper use of birth weights reference percentiles to classify birth weight is of great significance for clinical work and research.

Several countries, including Japan, Australia, South Korea, south India, Norway and the United States of America have developed population-based twin birth weight references to assist in accurately evaluating the growth of twins^7,9–13^. Findings in these countries have demonstrated the importance of the development of national birth weight standards for twins. Researchers have suggested that gestational age-specific birth weight reference percentiles should be updated every 5–10 years^1^. However, there is still no reference standard for twin birth weights in southern China.

The current study aims to construct the sex- and gestational age (week)-specific birth weight reference percentiles for twins born in southern China, stratified by placental chorionicity (monochorionic and dichorionic placentation).

## Materials and Methods

All birth data were obtained from the Guangdong Provincial Birth Certificate System between Jan 1st, 2014 and Dec 31st, 2017. The system covers more than 1900 medical institutions and collects all information about mothers and infants. After birth, maternity medical workers place newborn infants on electronic scales to obtain stable weight data (weighing accuracy is within 1 g). In some cases, health care attendants or midwives fill in the newborns’ information in the regional maternal and child information system. The system sets logic correction to ensure that the entered birth weight falls within a feasible range. Finally, regional maternal and child information are uploaded to the Guangdong Provincial Birth Certificate System. The Chief of Midwives and the Chief of Physicians in hospitals then confirm the information entered into the data system. Before the birth certificate is issued, the Department of Medical Administration and parents are also asked to confirm the birth information. All of the information is verified by medical professionals. The birth registry database includes the child’s date of birth, gestational age (week) at birth, birth weight, infant sex, parents’ ages, registered residence, method of delivery and placenta chorionicity, etc. From the database, we obtained 161,134 cases of twins. We excluded stillbirths (48 cases) and deaths within seven days (10 cases), which together accounted for about 0.04% (58 cases) in all twins. The final analytical sample included 161,076 twin births. Because this study is based on administrative data collected from a large population, it was not possible to obtain informed consents; however, the study was reviewed and approved by the Ethics Committee of Guangdong Women and Children Hospital.

We analyzed the raw data of all twin newborns (40,090 in 2014, 38,285 in 2015, 42,241 in 2016 and 40,460 in 2017). The gestational age (week) was determined by combining mother-reported last menstrual period, ultrasound examination, and postnatal gestational age (week) assessment. The chorionicity of the placenta was judged by ultrasound data collected during the first trimester (about 6~7 weeks of gestation) and confirmed by data collected during examination of the placenta after birth.

Birth weight percentiles were created by using the Lambda Mu Sigma (LMS) method, which were fit using the GAMLSS package, based on the assumption that birth weight had a Box-Cox Cole and Green (BCCG) distribution^14,15^. The GAMLSS method allows modeling of various kurtosis asymmetric distribution and the estimation of smooth percentiles to establish birth weight percentile curves for newborns of both genders. According to Cole’s reports^16^, a sample size of n >1000 is needed to use the GAMLSS technique to fit a curve. The Schwarz Bayesian criterion, which entails stricter curve smoothing, can be used to judge the pros and cons of the model, as well as to ensure the smoothness and accuracy of the model. GAMLSS is based on the LMS method with a specific distribution of (μ,σ,υ,τ). We used Box-Cox t (BCT) to model birth weight, a method that combines Box-Cox-Cole-Green (BCCG) with the Box-Cox-power-exponential (BCPE) distribution. Note that we take into account the skewness and kurtosis of the data to express the value of the predictor. In addition, we made the model residuals better modified and the shape of the curve tends to be smoother. Model selection was based on the generalized Akaike Information Standard (G-AIC). That is, we selected the model with the smallest GAIC value. The smoothed data were represented by birth weight percentile curves. The curves appeared in intervals of one gestational week. We estimated mean birth weights and corresponding standard deviations for twins at the 3rd, 5th, 10th, 25th, 50th, 75th, 90th, 95th, and 97th percentiles from 25 to 42 completed weeks based on the smoothed, estimated curves. The percentiles were estimated separately by infant sex (male and female) and by chorionicity. SGA and LGA were defined as birth weights below the 10^th^ or above the 90th percentile values at a given sex- and gestational week, respectively.

Next, we used twin birth weight data collected between Jan 1st, 2018 and Apr 30st, 2018, encompassing 12,371 twin births, to verify the reliability of the four standards. We accomplished this by calculating the SGA ratio and the LGA ratio according to the standards’ 10th and 90th percentile values. If standards are reliable, the gestational age (week)-specific SGA and LGA ratios should fluctuate around 10%. We also compared the SGA and LGA ratios we generated to those generated using birth weight references from Australia, South Korea and China (established based on a birth defects surveillance system). Since birth weight may differ by race and ethnicity, the birth weight standards from other countries may differ from those we produced. Moreover, given that birth weights in China may have changed since the implementation of the two-child policy in China in 2016, previously produced birth weight standards in China may be outdated. In both cases, this could result in inaccuracies in the classification of infants as SGA or LGA.

The GAMLSS package (version 5.0.6) for R statistical software (version 3.4.2) was used for analysis.

## Results

As showed in Table 1, a total of 83,940 pregnant women and 161,076 twin births included in analysis. Of the pregnant women, 55505 (66.2%) were 25 to 34 years-old and 2.0% were above age 40; 79,716 (95.0%) mothers were members of the Han ethnic group and 61,768 (73.6%) mothers were multipara. Vaginal delivery and cesarean section delivery accounted for 18.6% and 59.2% of all births respectively, while the remaining delivery modes were unclear. Of the twin births, 84,208 (52.3%) were male twins and 76868 (47.7%) were female twins. Of the 98,111 twin births which chorionic placentation were known, 34,338 were monochorionic male twins, 31,567 were monochorionic female twins, 16,720 were dichorionic male twins and 15,486 were dichorionic female twins. The mean birth weights and associated standard deviations (SD) for male twins with monochorionic and dichorionic placentation were (2436 ± 453) g and (2506 ± 480) g, respectively. While the mean birth weights and associated standard deviations (SD) of female twins with monochorionic and dichorionic placentation were (2361 ± 423) g and (2400 ± 459) g, respectively. Premature twins born at 28–36 weeks and term twins born at ≥37 weeks accounted for 45.9% and 53.7% of all twins, respectively. Low birth weight twin births (birth weight <2500 g) and normal birth weight twin births (birth weight ≥2500 g) accounted for 52.2% and 47.8% of all twins, respectively.

**Table 1:** Maternal and neonatal characteristics of twin births in this study (2014–2017).

Variables	Male			Female			Total		
	Monochorionic	Dichorionic	Total*	Monochorionic	Dichorionic	Total*	Monochorionic	Dichorionic	Total*
**Number of mothers**	17384	8830	44130	15946	7952	39810	33330	16782	83940
**Maternal age (years)**									
≤20	835(4.8)	200(2.3)	1327(3.0)	906(5.7)	175(2.2)	1408(3.5)	1741(5.2)	375(2.2)	2731(3.1)
21–25	4383(25.2)	1482(16.8)	8278(18.8)	4157(26.1)	1376(17.3)	7668(19.3)	8540(25.6)	2858(17.0)	15947(19.0)
26–30	6543(37.6)	3459(39.2)	16428(37.2)	5922(37.1)	3146(39.6)	15026(37.8)	12465(37.4)	6605(39.4)	31454(37.5)
31–35	4038(23.2)	2669(30.2)	12850(29.1)	3584(22.5)	2343(29.5)	11199(28.1)	7622(22.9)	5012(29.9)	24051(28.7)
36–40	1344(7.7)	880(10.0)	4294(9.7)	1199(7.5)	794(10.0)	3798(9.5)	2543(7.6)	1674(10.0)	8093(9.6)
41–45	213(1.2)	112(1.3)	722(1.6)	164(1.0)	99(1.3)	594(1.5)	377(1.1)	211(1.3)	1316(1.6)
≥46	28(0.2)	28(0.3)	231(0.5)	14(0.1)	19(0.2)	117(0.3)	42(0.1)	47(0.3)	348(0.4)
**Maternal ethnicity**									
Han	16816(96.7)	8469(95.9)	41855(95.0)	15434(96.8)	7666(96.4)	37857(95.1)	32250(96.8)	16135(96.1)	79716(95.0)
Minorities	568(3.3)	361(4.1)	2275(5.0)	512(3.2)	286(3.6)	1949(4.9)	1080(3.2)	647(3.9)	4224(5.0)
**Parity**									
Nulliparous	3951(22.7)	2373(26.9)	11461(26.0)	3806(23.9)	2140(26.9)	10710(26.9)	7757(23.3)	4513(26.9)	22172(26.4)
Parous	13433(77.3)	6457(73.1)	32669(74.0)	12140(76.1)	5812(73.1)	29096(73.1)	25573(76.7)	12269(73.1)	61768(73.6)
**Method of delivery**									
Caesarean section	9912(57.0)	5215(59.1)	26335(59.7)	8794(55.2)	4624(58.2)	23348(58.7)	18706(56.1)	9839(58.6)	49684(59.2)
Virginal	3297(19.0)	1095(12.4)	7986(18.1)	3440(21.6)	1028(12.9)	7666(19.3)	6737(20.2)	2123(12.7)	15654(18.6)
Un-know	4175(24.0)	2520(28.5)	9809(22.2)	3712(23.3)	2300(28.9)	8792(22.1)	7887(23.7)	4820(28.7)	18602(22.2)
**Number of newborns**	34338	16720	84208	31567	15486	76868	65905	32206	161076
**Gestational age (weeks)**									
25–27	102(0.3)	68(0.4)	372(0.4)	45(0.1)	46(0.3)	238(0.3)	147(0.2)	114(0.4)	610(0.4)
28–32	1976(5.8)	940(5.6)	5160(6.1)	1454(4.6)	833(5.4)	4075(5.3)	3431(5.2)	1773(5.5)	9235(5.7)
33–36	13160(38.3)	6347(38.0)	34426(40.9)	11415(36.2)	5740(37.1)	30275(39.4)	24575(37.3)	12087(37.5)	64701(40.2)
37–42	19100(55.6)	9365(56.0)	44250(52.5)	18653(59.1)	8867(57.3)	42280(55.0)	37753(57.3)	18232(56.6)	86530(53.7)
**Birth weight (g)**									
Mean ± SD	2436 ± 453	2506 ± 480	2457 ± 475	2361 ± 423	2400 ± 459	2373 ± 450	2400 ± 441	2455 ± 473	2417 ± 465
<1500	1094(3.2)	481(2.9)	2999(3.6)	995(3.2)	549(3.5)	2926(3.8)	2089(3.2)	1030(3.2)	5925(3.7)
1500–1999	3852(11.2)	1525(9.1)	8855(10.5)	4171(13.2)	1835(11.9)	9911(12.9)	8023(12.2)	3360(10.4)	18766(11.7)
2000–2499	11893(34.6)	5311(31.8)	28541(33.9)	13048(41.3)	6012(38.8)	30876(40.2)	24941(37.8)	11323(35.2)	59417(36.9)
2500–2999	13954(40.6)	7016(42.0)	34028(40.4)	11406(36.1)	5760(37.2)	27538(35.8)	25360(38.5)	12776(39.7)	61566(38.2)
≥3000	3545(10.3)	2387(14.3)	9785(11.6)	1947(6.2)	1330(8.6)	5617(7.3)	5492(8.3)	3717(11.5)	15402(9.6)

^*^Total: include monochorionic, dichorionic, and un-know chorionic placentation.

**Figure 1:**
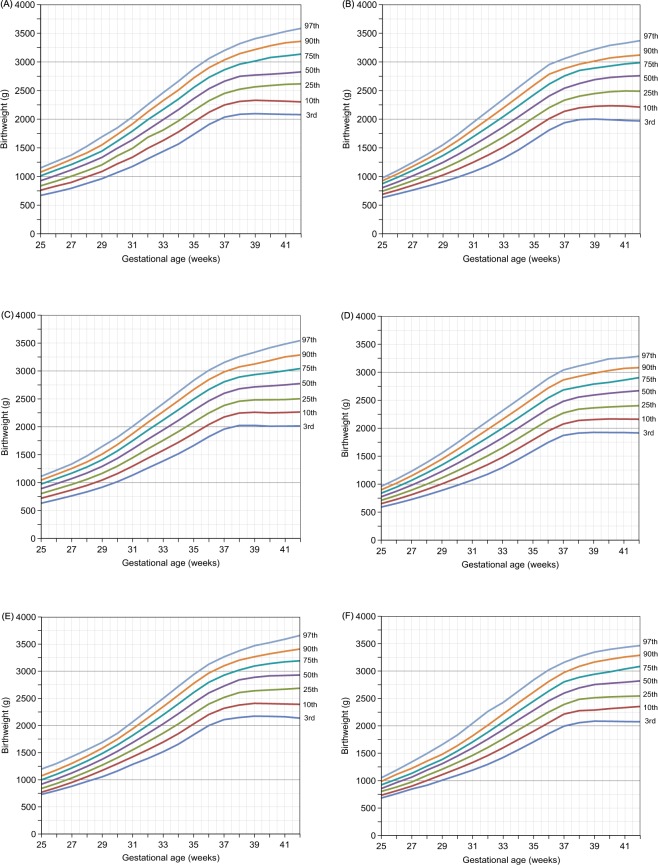
Smoothed percentiles of birth weight (gms) by gestational weeks for: (**A**) overall male twins; (**B**) overall female twins; (**C**) monochorionic male twins; (**D**) monochorionic female twins; (**E**) dichorionic male twins; and (**F**) dichorionic female twins.

Table 2 displays smoothed percentiles for birth weights by gestational age (week) for male and female twins. We next grouped all monochorionic twins based on gestational age (week) and present the resulting data at the 3^rd^,10^th^,25^th^,50^th^,75^th^,90^th^, and 97^th^ percentiles in Table 3. Dichorionic twins were plotted in the same way, with Table 4 displaying smoothed percentiles for birth weights (in grams) of dichorionic male twins and dichorionic female twins. As the gestational age (week) increases, the growth curves for various percentiles become smoother and increasingly steadily. In the 10th, 50th, and 90th percentile graphs of monochorionic twins and dichorionic twins, male twins showed higher BWs than females in the total infant graphs at each GA. Twins showed the most weight gain at 34–35 weeks, with growth slowing after 38 weeks (Fig. 1). Table 4 provides the sex-specific proportions of births at 25–42 gestational weeks.

**Table 2:** Smoothed percentiles for birth weight (g) of male and female twins.

GA (weeks)	Male twin babies smoothed percentiles										Female twin babies smoothed percentiles									
	*N*	*C3*	*C10*	*C25*	*C50*	*C75*	*C90*	*C97*	*Mean*	*SD*	*N*	*C3*	*C10*	*C25*	*C50*	*C75*	*C90*	*C97*	*Mean*	*SD*
25	34	670	761	837	926	1011	1077	1150	924	214	35	632	689	745	805	875	926	975	813	231
26	117	730	832	918	1017	1112	1184	1262	1021	238	75	696	765	833	904	986	1046	1105	901	241
27	221	794	897	1004	1114	1208	1298	1372	1123	256	130	763	847	927	1012	1106	1177	1247	1008	248
28	421	878	993	1099	1219	1322	1410	1524	1232	273	332	833	933	1027	1125	1232	1314	1395	1124	293
29	597	962	1087	1203	1333	1443	1551	1692	1340	292	433	908	1026	1135	1247	1367	1462	1554	1249	290
30	863	1069	1220	1360	1492	1616	1733	1848	1489	324	709	991	1131	1258	1388	1523	1632	1740	1385	322
31	1193	1176	1337	1492	1641	1794	1918	2042	1648	346	989	1084	1250	1396	1544	1696	1821	1947	1549	333
32	2091	1310	1494	1685	1816	1992	2129	2257	1811	369	1617	1192	1378	1540	1704	1871	2010	2151	1705	360
33	3146	1439	1628	1810	1992	2170	2325	2463	1996	382	2776	1319	1518	1694	1871	2051	2202	2355	1874	379
34	5451	1567	1779	1971	2168	2352	2510	2664	2175	400	4685	1468	1674	1858	2047	2239	2399	2561	2051	396
35	8745	1737	1952	2153	2360	2555	2722	2880	2364	423	7767	1639	1843	2032	2229	2430	2597	2763	2235	404
36	17097	1910	2123	2324	2534	2732	2901	3062	2527	421	15071	1811	2011	2203	2406	2614	2787	2955	2410	412
37	24503	2036	2249	2451	2663	2863	3036	3200	2674	427	22086	1938	2138	2332	2541	2755	2884	3057	2539	417
38	11127	2086	2311	2524	2749	2961	3145	3319	2745	466	11003	1993	2199	2403	2624	2852	2961	3146	2618	446
39	4420	2097	2330	2565	2771	3015	3216	3406	2779	523	4717	2002	2225	2448	2691	2892	3015	3222	2695	507
40	3810	2090	2322	2588	2785	3077	3283	3467	2792	535	4055	1991	2234	2479	2728	2928	3069	3289	2732	516
41	344	2084	2314	2608	2803	3105	3334	3532	2820	540	376	1978	2229	2493	2747	2964	3097	3327	2752	556
42	56	2080	2302	2617	2825	3136	3359	3585	2832	535	42	1969	2211	2489	2758	2987	3120	3371	2761	540

**Table 3:** Smoothed percentiles for birth weight (g) of monochorionic male and female twins.

GA (weeks)	Monochorionic male twin babies smoothed percentiles										Monochorionic female twin babies smoothed percentiles									
	*N*	*C3*	*C10*	*C25*	*C50*	*C75*	*C90*	*C97*	*Mean*	*SD*	*N*	*C3*	*C10*	*C25*	*C50*	*C75*	*C90*	*C97*	*Mean*	*SD*
25	12	631	720	804	892	974	1046	1113	895	204	14	591	652	711	775	839	899	961	780	194
26	29	696	793	885	980	1071	1148	1222	983	248	15	656	728	798	873	947	1017	1089	870	272
27	71	763	868	967	1071	1169	1253	1333	1070	263	26	727	812	894	981	1068	1149	1232	983	277
28	151	835	950	1059	1172	1279	1370	1478	1170	285	92	805	905	999	1100	1201	1293	1388	1105	286
29	212	918	1046	1166	1291	1409	1511	1647	1293	266	150	889	1004	1113	1229	1343	1447	1555	1224	318
30	319	1017	1161	1295	1435	1568	1681	1813	1432	306	213	978	1112	1237	1369	1498	1616	1738	1372	318
31	428	1134	1295	1446	1603	1751	1878	2008	1606	351	355	1073	1226	1368	1518	1664	1796	1932	1516	340
32	866	1262	1439	1605	1777	1940	2080	2213	1780	355	645	1176	1347	1505	1670	1830	1975	2123	1669	350
33	1264	1387	1578	1757	1944	2121	2272	2416	1943	390	1085	1297	1482	1652	1830	2002	2157	2313	1828	378
34	2103	1517	1722	1915	2115	2305	2468	2623	2118	396	1798	1439	1632	1811	1998	2179	2340	2503	1996	388
35	3437	1664	1879	2082	2295	2495	2668	2831	2289	435	3022	1592	1791	1978	2174	2362	2530	2697	2176	398
36	6350	1824	2042	2248	2465	2669	2846	3013	2458	430	5501	1746	1949	2142	2346	2542	2717	2889	2342	416
37	9597	1959	2176	2383	2600	2806	2984	3153	2606	430	8432	1871	2075	2271	2480	2683	2862	3039	2478	415
38	5626	2025	2247	2459	2681	2892	3074	3258	2687	446	5690	1913	2138	2339	2555	2737	2925	3110	2559	430
39	2402	2025	2260	2482	2715	2935	3125	3335	2714	474	2727	1925	2154	2363	2592	2788	2981	3171	2598	466
40	1256	2010	2250	2484	2732	2966	3187	3416	2729	514	1572	1923	2165	2379	2623	2818	3029	3238	2621	494
41	193	2012	2256	2488	2751	3004	3253	3483	2756	533	213	1922	2163	2391	2648	2859	3068	3257	2643	510
42	28	2016	2265	2503	2775	3044	3287	3545	2770	517	20	1915	2160	2402	2670	2903	3082	3285	2672	503

**Table 4:** Smoothed percentiles for birth weight (g) of dichorionic male and female twins.

GA (weeks)	Dichorionic male twin babies smoothed percentiles										Dichorionic female twin babies smoothed percentiles									
	*N*	*C3*	*C10*	*C25*	*C50*	*C75*	*C90*	*C97*	*Mean*	*SD*	*N*	*C3*	*C10*	*C25*	*C50*	*C75*	*C90*	*C97*	*Mean*	*SD*
25	15	730	770	839	918	998	1072	1197	920	192	16	683	734	803	855	923	986	1052	850	196
26	26	802	856	931	1016	1102	1181	1300	1018	221	17	762	815	883	965	1026	1114	1192	967	211
27	35	880	951	1033	1125	1218	1302	1426	1122	261	25	847	900	975	1064	1133	1224	1341	1071	217
28	87	969	1057	1147	1246	1345	1435	1554	1256	254	70	916	1003	1096	1192	1265	1356	1495	1193	279
29	100	1055	1173	1271	1378	1485	1582	1690	1374	277	86	1008	1110	1208	1309	1386	1480	1658	1315	238
30	146	1162	1296	1406	1525	1642	1749	1857	1530	335	140	1098	1218	1331	1449	1541	1639	1833	1456	302
31	230	1285	1423	1551	1686	1818	1940	2067	1685	311	186	1192	1332	1462	1597	1706	1819	2048	1598	295
32	378	1393	1555	1702	1855	2004	2142	2285	1862	366	351	1298	1459	1606	1759	1885	2013	2264	1763	362
33	574	1516	1694	1858	2029	2196	2346	2499	2035	356	505	1423	1600	1763	1931	2073	2216	2427	1938	359
34	1023	1656	1849	2029	2217	2399	2560	2721	2221	405	913	1563	1750	1926	2109	2264	2420	2632	2114	398
35	1635	1828	2028	2217	2416	2607	2775	2938	2422	409	1456	1708	1902	2087	2283	2451	2616	2838	2287	392
36	3100	1999	2202	2395	2600	2796	2966	3129	2610	422	2858	1859	2059	2252	2458	2636	2811	3022	2463	419
37	5129	2108	2317	2517	2727	2927	3100	3265	2731	426	4815	1995	2215	2391	2597	2803	2974	3159	2595	417
38	2551	2147	2377	2607	2841	3022	3204	3376	2836	472	2404	2058	2277	2485	2693	2886	3086	3264	2687	447
39	980	2172	2408	2640	2888	3096	3267	3470	2893	525	952	2086	2288	2513	2754	2942	3165	3346	2763	521
40	614	2168	2403	2655	2915	3140	3321	3527	2912	599	599	2083	2314	2528	2776	2985	3214	3395	2778	579
41	77	2161	2396	2669	2922	3173	3367	3590	2924	544	77	2078	2333	2537	2796	3038	3257	3433	2799	578
42	22	2135	2390	2687	2930	3192	3409	3660	2933	582	18	2075	2354	2545	2820	3085	3288	3465	2824	563

**Figure 2:**
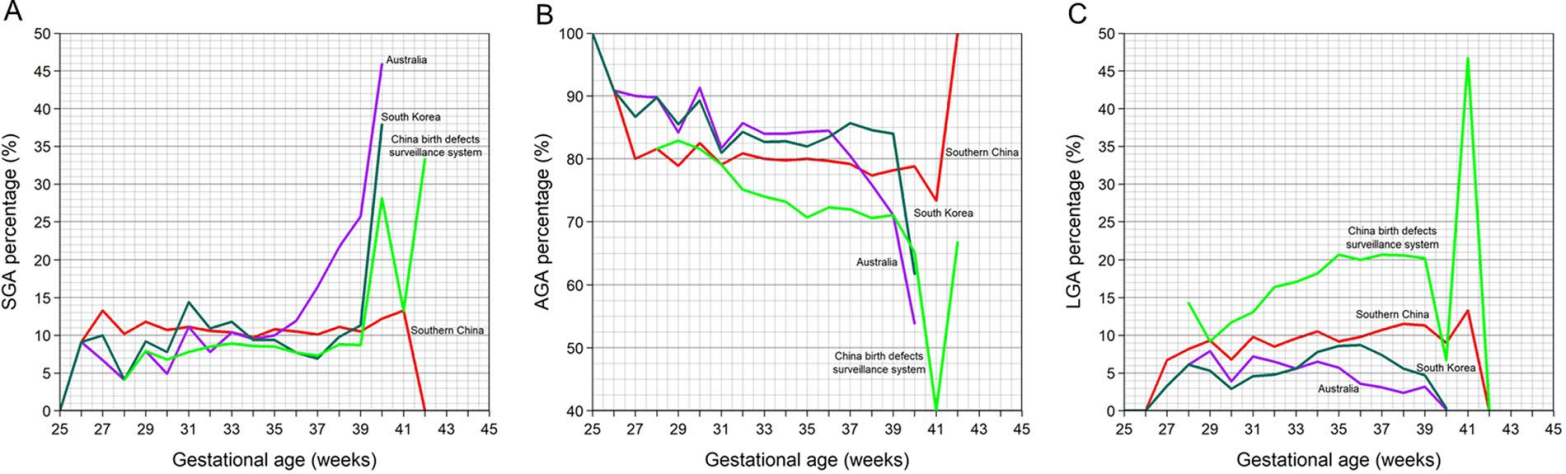
Comparison with the birth weight references in Australia, South Korea, and China (based on the birth defects surveillance system standards). (**A**) At each gestational age (week), the SGA rate is calculated by dividing the number of twins who are defined as SGA by the total number of twins born during this gestational age (week). (**B**) Appropriate for gestational age (AGA) twins were defined as those with birth weights falling within the 10^th^ and 90^th^ percentiles. The AGA rate is calculated by dividing the number of twins who are defined as AGA by the total number of twins born during the gestational age (week) (**C**) The LGA rate is calculated by dividing the number of twins who are defined as LGA by the total number of twins born during the gestational age (week).

Table 5 provides the SGA and LGA ratios of four standards. The curves showing the incidence of SGA at different gestational ages were used to produce criteria, which were then compared to the previous criteria in China, as well as the criteria from Australia and South Korea (Fig. 2). Since Australia and South Korea’s standards only cover gestational ages ranging from 25–40 weeks, we only use these references to calculate SGA and LGA at 25–40 weeks. Moreover, the China birth defects surveillance system standards only cover gestational ages between 28–42 weeks. As a result, we only use this reference to calculate SGA and LGA at 28–42 weeks. As expected, the thresholds derived from Australia standards captured a greater proportion of SGA births (45.9%) in 40 gestational age (week), while included only 4.1% in 28 gestational age (week) among the gestation ranges in their research dataset. On the other hand, the thresholds derived from South Korea standards below the 10th and above the 90th percentile across all gestational age (week) categories were from 3.3% to 37.9%. The thresholds derived from China birth defects surveillance system standards captured a greater proportion of LGA births (46.7% in 41 gestational age (week)), while included only 6.7% (40 gestational age (week)) within the gestation ranges in their research dataset. In our research, the 10th and 90th-percentile proportions of birth weight for gestational week which got by Birth weight percentiles of southern China were relatively stable. The maximum value was found in SGA of 27 and 41 gestational age (week) (13.3%), while the minimum value is found in LGA of 27 gestational age (week) (6.7%).

**Table 5:** SGA and LGA ratios of four standards.

GA (weeks)	*N*	southern China			Australia			South Korea			China*		
		SGA	AGA	LGA	SGA	AGA	LGA	SGA	AGA	LGA	SGA	AGA	LGA
25	7	0	100	0	0	100	0	0	100	0			
26	11	9.1	90.9	0	9.1	90.9	0	9.1	90.9	0			
27	30	13.3	80	6.7	6.7	90	3.3	10	86.7	3.3			
28	49	10.2	81.6	8.2	4.1	89.8	6.1	4.1	89.8	6.1	4.1	81.6	14.3
29	76	11.8	78.9	9.3	7.9	84.2	7.9	9.2	85.5	5.3	7.9	82.9	9.2
30	103	10.7	82.5	6.8	4.9	91.3	3.9	7.8	89.3	2.9	6.8	81.6	11.7
31	153	11.1	79.1	9.8	11.1	81.7	7.2	14.4	81	4.6	7.8	79.1	13.1
32	293	10.6	80.9	8.5	7.8	85.7	6.5	10.9	84.3	4.8	8.5	75.1	16.4
33	450	10.4	80	9.6	10.4	84	5.6	11.8	82.7	5.6	8.9	74	17.1
34	873	9.7	79.8	10.5	9.5	84	6.5	9.4	82.8	7.8	8.6	73.2	18.2
35	1288	10.8	80	9.2	10	84.3	5.7	9.4	82	8.6	8.5	70.7	20.7
36	2461	10.5	79.7	9.8	11.9	84.5	3.6	7.7	83.5	8.7	7.7	72.3	20
37	3322	10.1	79.2	10.7	16.4	80.5	3.1	6.9	85.7	7.4	7.3	72	20.7
38	1391	11.1	77.4	11.5	21.7	75.9	2.4	9.8	84.6	5.6	8.8	70.6	20.6
39	494	10.5	78.2	11.3	25.7	71.1	3.2	11.3	84	4.7	8.7	71.1	20.2
40	1352	12.2	78.8	9	45.9	53.9	0.2	37.9	61.8	0.4	28.1	65.2	6.7
41	15	13.3	73.4	13.3							13.3	40	46.7
42	3	0	100	0							33.3	66.7	0

^*^Based on the birth defects surveillance system.

## Discussion

In this study, we constructed new birth weight percentage curves for twins born in southern China. We have estimated percentage curves separately by chorionicity in order to account for chorionic membranes during twin births. Our comparison of percentile curves by chorionicity showed that birth weights of dichorionic twins were higher than monochorionic twins at 25 to 42 weeks of gestation. This finding is consistent with research conducted in south India and the US^7,17^. The low birth weight of monochorinic twins can be attributed to a reduction in weight due to a shared placenta, as well as to reduce effectiveness of the placenta^18^.

The average birth weight of male infants is greater than that of female infants for both monochorionic twins and dichorionic twins. The overall pattern of change in birth weight over gestational age is characterized by a rapid increase in birth weight up until week 37, followed by a reduced rate of change afterward. Both male and female infants grew at the fastest rate between 34 and 35 weeks, gaining an average of 192 g and 182 g per week, respectively. Studies of twin pregnancies in the US have found that twin infants have the fastest weight gain between 32 weeks and 34 weeks^17^, while the East Flanders Prospective Twin Survey (EFPTS) found that the most rapid period of infant weight gain occurred between 32 weeks and 34 weeks, with 156 g gained per week^19^.

Because of improvement in medical care facilities and nutrition in China, the proportion of twins with fetal growth restriction has declined, while perinatal survival has improved. Furthermore, due to the large sample size used in our analysis, the birth weight standard we have produced can shed new light on the current situation of twins in southern China.

According to the twin birth weight standard we have constructed for southern China, the highest prevalence (13.3%) of SGA was observed at 41 weeks of gestation, while the lowest (9.1%) was observed at 26 weeks of gestation. Relative to the other three standards, our standard led to a more stable estimate of the prevalence of LGA, which ranged from 6.7% to 11.5%. If we were to instead use one of the other three standards, we would likely misclassify SGA and LGA across all gestational age groups. In particular, the other standards lead to very different estimates of the SGA rate between 39 and 40 weeks of gestation. Compared to southern China’s twin birth weight standard, a smaller number of twins were classified as LGA by the Australian and Korean standards. However, a larger number of twins were defined as LGA if we were to use the Chinese standard (based on birth defects surveillance system). This suggests that twin growth standards for healthy twins developed in other countries are not applicable to the population of southern China. Moreover, it is important to regularly update the reference, in order to identify changes in birth weight distributions of twins over time. In addition, previous research has suggested that chrionicity should be taken into account when assessing twin fetal development^17^. In particular, fetal growth appears to differ for twins with monochorionic and dichorionic placentation. Until now, classification of chorionicity was not established for twin birth weight standards in southern China.

Due to the lack of appropriate reference tools, birth weight percentiles for singletons are commonly used in clinical practice in China. In this study, the use of a large, nationally representative population-based sample of twins ensures a more representative and accurate estimate of percentiles.

Unfortunately, we did not collect data on environmental factors that may have affected the pregnant women and fetuses in the study, including socio-economic conditions, diet or nutritional status. Therefore, we cannot directly analyze the relationship between environmental factors and birth weight distributions. Secondly, as with other population-based studies, our data are based on birth registry data, rather than longitudinal measurement of the development of the same fetuses over the course of pregnancy. That is, we have not measured in utero fetal growth. Birth weight percentiles are not the same as intrauterine growth percentiles in that birth weight percentiles do not reflect fetal growth but rather size at birth. The birth weight of premature babies may be affected by the pathological process leading to premature birth and the developmental status during the period of extrauterine growth to full term may be different from that of intrauterine growth until full term^20,21^. It has been suggested that preterm births should be assessed using estimated utero fetal growth trajectories rather than birth weight percentile, given that preterm neonates are likely affected by fetal growth restriction^20^. However, it is difficult to estimate utero fetal growth weight, due to challenges in obtaining accurate measurements, including estimates for fetal weight calculations and the formulas needed for the calculations^21^.

## References

[1] Zhang, B. *et al*. Birth weight percentiles for twin birth neonates by gestational age in China. *Sci rep-uk* **6**(1), 10.1038/srep31290 (2016). 10.1038/srep31290 PMC4978964 27506479

[2] Cheong-See F (2016). Prospective risk of stillbirth and neonatal complications in twin pregnancies: systematic review and meta-analysis. BMJ.

[3] Team NHAF, Limei J, Shanshan L, Caixia L, Chong Q (2017). Ultrasound Examination of Twin Pregnancy Technical Specifications (2017). Chinese Journal of Practical Gynecology and Obstetrics.

[4] Sherer DM (2001). Adverse perinatal outcome of twin pregnancies according to chorionicity: review of the literature. Am J Perinatol.

[5] Saenger P, Czernichow P, Hughes I, Reiter EO (2007). Small for gestational age: short stature and beyond. Endocr rev.

[6] Weight, B. Large for Gestational Age. (Alphascript Publishing, 2010).

[7] Premkumar, P. *et al*. Birth weight centiles by gestational age for twins born in south India. *Bmc pregnancy childb* **1**6(1), 10.1186/s12884-016-0850-y (2016). 10.1186/s12884-016-0850-y PMC4806424 27012538

[8] Lipecka-Kidawska E, Gottwald L, Niewiadomska-Jarosik K (2005). Intrauterine growth restriction. Int J Gynaecol Obstet.

[9] Kato N, Uchiyama Y (2005). Reference birth-length range for multiple-birth neonates in Japan. J Obstet Gynaecol Res.

[10] Li Z, Umstad MP, Hilder L, Xu F, Sullivan EA (2015). Australian national birth weight percentiles by sex and gestational age for twins, 2001–2010. Bmc pediatr.

[11] Lim JS (2014). New Korean reference for birth weight by gestational age and sex: data from the Korean Statistical Information Service (2008–2012). Annals of Pediatric Endocrinology & Metabolism.

[12] Glinianaia SV, Skjaerven R, Magnus P (2000). Birth weight percentiles by gestational age in multiple births. A population-based study of Norwegian twins and triplets. Acta Obstet Gynecol Scand.

[13] Mikolajczyk RT (2011). A global reference for fetal-weight and birth weight percentiles. Lancet.

[14] Platt RW (2001). Detecting and eliminating erroneous gestational ages: a normal mixture model. Stat med.

[15] Mikis D. S., Robert A. R., Gillian Z. H., V. V. & B. F. D. Centile estimation in Flexible Regression and Smoothing Using GAMLSS in R. 449–480 (Chapman & Hall/CRC, 2017).

[16] Cole TJ (2009). Age- and size-related reference ranges: a case study of spirometry through childhood and adulthood. Stat med.

[17] Ananth CV, Vintzileos AM, Shen-Schwarz S, Smulian JC, Lai YL (1998). Standards of birth weight in twin gestations stratified by placental chorionicity. Obstet gynecol.

[18] Ramos-Arroyo MA, Ulbright TM, Yu PL, Christian JC (1988). Twin study: relationship between birth weight, zygosity, placentation, and pathologic placental changes. Acta Genet Med Gemellol (Roma).

[19] Loos, R. J. *et al*. Determinants of birth weight and intrauterine growth in liveborn twins. *Paediatric & Perinatal Epidemiology*, **19**(s1), 15–22 (2005). 10.1111/j.1365-3016.2005.00611.x 15670117

[20] Cooke RW (2007). Conventional birth weight standards obscure fetal growth restriction in preterm infants. Arch Dis Child Fetal Neonatal Ed.

[21] Ehrenkranz RA (2007). Estimated fetal weights versus birth weights: should the reference intrauterine growth curves based on birth weights be retired?. Arch Dis Child Fetal Neonatal Ed.

